# Antibacterial and Anti-Inflammatory Effects of Novel Peptide Toxin from the Spider *Pardosa astrigera*

**DOI:** 10.3390/antibiotics9070422

**Published:** 2020-07-19

**Authors:** Min Kyoung Shin, In-Wook Hwang, Yunkyung Kim, Seung Tae Kim, Wonhee Jang, Seungki Lee, Woo Young Bang, Chang-Hwan Bae, Jung-Suk Sung

**Affiliations:** 1 Department of Life Science, Donnguk University-Seoul, Biomedi Campus, 32, Dongguk-ro, Ilsandong-gu, Goyang-si 10326, Gyeonggi-do, Korea; samantha1994@naver.com (M.K.S.); hiw9100@gmail.com (I.-W.H.); 252114@naver.com (Y.K.); wany@dongguk.edu (W.J.); 2 Life and Environment Research Institute, Konkuk University, 120, Neungdong-ro, Gwangjin-gu, Seoul 05029, Korea; stkim2000@hanmail.net; 3 Biological and Genetic Resources Assessment Division, National Institute of Biological Resources, 42, Hwangyeong-ro, Seo-gu, Incheon 22689, Korea; metany@korea.kr (S.L.); wybang@korea.kr (W.Y.B.); bae0072@korea.kr (C.-H.B.)

**Keywords:** *Pardosa astrigera*, spider venom gland, transcriptome, in silico analysis, antibacterial peptide, anti-inflammation

## Abstract

The prevalence of antibiotic-resistant bacteria has become an immediate threat to public health. Antimicrobial peptides are attracting attention as a new source of antibiotics due to their ability to prevent drug-resistances with fewer side effects. Spider venom is composed of various bioactive substances with multiple functionalities such as antimicrobial and anti-inflammatory effects. Here, RNA sequencing was conducted on the venom gland of the spider *Pardosa astrigera,* and a potential toxin peptide with antibacterial properties was selected via homology and in silico analysis. A novel toxin, Lycotoxin-Pa4a, inhibited both gram-negative and gram-positive bacteria by disrupting the outer and bacterial cytoplasmic membrane. Moreover, the peptide downregulated the expression of pro-inflammatory mediators while upregulating the level of anti-inflammatory cytokine by inactivating mitogen-activated protein kinase signaling in a lipopolysaccharide-stimulated murine macrophage cell line. In this research, we identified a novel peptide toxin, Lycotoxin-pa4a, with antibacterial and anti-inflammatory properties, suggesting its potential for the development of a new antibiotics, as well as offering insights into the utilization of biological resources.

## 1. Introduction

Antibiotics have dramatically reduced the risk of infection, contributing to public health since the discovery of penicillin by Alexander Fleming. However, due to the overuse of antibiotics and the limitation of their availability, drug-resistant bacteria have emerged and become an immediate threat to humankind worldwide [1,2]; there is therefore an urgent need to identify a new source of antibacterial agents.

Peptides possess pharmaceutical applicability owing to their high efficacy and selectivity towards cellular components. Owing to their chemical and physiological nature, the utilization and optimization of peptides can facilitate rapid drug designing. Moreover, combined treatment using conventional medication with peptides has reportedly demonstrated synergistic effects on pathological phenomena such as infectiona, cancera, and neurodegeneration [3,4,5]. As the biological value of peptides increases, the identification of novel peptides and investigation of their functionalities are gaining more interest.

The discovery of functional compounds starts with the screening of existing chemicals or natural substances. Animal venom is used for both defense and predation; it typically evolved in a complex manner along with the evolution of the animals themselves. Notably, spider venom consists of small ions, peptide, and proteins, indicating the presence of various bioactive compounds. Several studies have revealed that antimicrobial peptides (AMPs) are often located in venom glands of venomous species, and can protect the host from infections as well as serving several other functionalities [6,7]. For example, lycosin-I was isolated from the spider venom of *Lycosa singoriensis* via reverse-phase high-performance liquid chromatography (HPLC); it was shown to inhibit the growth of both bacteria and fungi and exhibited antiparasitic activity [8,9]. Latarcins, toxins from the *Lachesana tarabaevi* venom, were identified to be AMPs with cytolytic activity [10]. Despite their high specificity and selectivity, only limited sources of spider venom have been studied, leaving numerous peptides to be discovered. 

Next-generation sequencing (NGS) technology is attracting attention for use in the identification of physiologically active compounds from a biological origin, especially animal venom. The NGS technique enables high-throughput and effective attainment of an organism’s transcriptome, in concert with the technological development of RNA sequencing and de novo assembly [11]. It facilitates the construction of large transcriptomes in the absence of reference sequences when only a small amount of sample is available, making it easier to harness functional materials from RNA transcripts. Moreover, it is possible to predict functional substances through homology and structural analyses based on transcript information and databases of known biologically-derived materials [12,13].

In the present study, the transcriptome and functional peptide of *Pardosa astrigera (P. astrigera),* a species of spider indigenous to Korea, were analyzed. The peptide with potential functionality, Lycotoxin-Pa4a, was selected by performing a comparative analysis of homology and structural characteristics with known toxin peptides. The peptide was tested for antibacterial activity against gram-negative and gram-positive bacteria, and its mechanism of action was also investigated. Additionally, the anti-inflammatory effect of Lycotoxin-Pa4a was studied along with its underlying molecular pathway based on the immunomodulatory potential of the peptide. Our results suggested the discovery of a novel peptide possessing antibacterial and anti-inflammatory activities from transcripts of the spider venom.

## 2. Materials and Methods 

### 2.1. Sample Preparation

A *P. astrigera* specimen was collected from Suwon, Gyeonggi-do, Korea. The venom glands of the spider were separated from the chelicerae and stored after washing in phosphate buffered saline. TRIzol reagent (Life Technologies, Grand Island, NY, USA) was used for the extraction of total RNA, and subsequent RNA sequencing was performed at the Theragen Etex Bio institute (Suwon, Korea). The peptide [AMMAESRKDNCIPKHHECTSRPKDCCKQNLMQFKCSCMTIIDKNNKETERCKCDNSIFQKVAKTSVNIGKAVVTK] was synthesized by Komabiotech (Seoul, Korea) with a purity >97% and verified via mass spectroscopy and HPLC. The quality control result for the synthesized peptide is shown in Supplementary Figure S1. 

### 2.2. Bacterial Strains and Cell Lines 

All strains were purchased from the Korean Culture Center of Microorganisms (KCCM, Seoul, Korea) or the American Type Culture Collection (ATCC, Manassas, VA, USA). The following bacterial strains were used in this study: gram-negative bacteria *Escherichia coli* KCCM 11234 (*E. coli*) and *Pseudomonas aeruginosa* ATCC 9027 (*P. aeruginosa*) and gram-positive bacteria *Bacillus cereus* KCCM 21366 (*B. cereus*) and *Staphylococcus aureus* KCCM 11335 (*S. aureus*). Bacterial strains were cultured overnight at 37 °C on tryptic soy agar (TSA, Difco Laboratories, Detroit, MI, USA) plates and subsequently inoculated in tryptic soy broth (TSB, Difco Laboratories) at 37 °C for 18 h under shaking and incubation. The murine macrophage cell line, RAW 264.7, was obtained from ATCC and cultured in Dulbecco’s modified eagle medium (Gibco, Grand Island, NY, USA) supplemented with 10% fetal bovine serum (Gibco) and 1% penicillin and streptomycin (Gibco). The cells were maintained under humidified air with 5% CO_2_ at 37 °C.

### 2.3. Antibacterial Activity Assay 

A colony-forming unit (CFU) assay was performed using gram-negative and gram-positive bacteria to determine antibacterial activity. Each strain was grown to mid-log phase and diluted to 2 × 10^5^ CFU/mL. Lycotoxin-Pa4a was serially diluted and mixed with an equal volume of bacteria. After 3 h incubation, the samples were spread onto TSA plates and incubated overnight at 37 °C. Relative colony formation was measured by counting colonies on each plate, and was expressed as a percentage compared with the extent of colony formation on the control plate.

### 2.4. Membrane Permeability Test 

The permeabilization of the bacterial outer membrane was determined by 1-N-phenylnaphthylamine (NPN, Sigma-Aldrich, St. Louis, MO, USA) uptake assay. The cultured cells were resuspended in 5 mM 4-(2-Hydroxyethyl)piperazine-1-ethanesulfonic acid (HEPES, Sigma-Aldrich) buffer at a concentration of 1 × 10^8^ CFU/mL and then mixed with the synthesized peptide and NPN at final concentrations of 4 μM and 10 μM, respectively. The 200-μL samples were transferred to a black 96-well microplate, and the fluorescence was measured for 10 min (excitation: 350 nm; emission: 420 nm) [14]. The depolarization of the bacterial cytoplasmic membrane by the peptide was evaluated using 3, 3′-dipropylthiadicarbocyanine iodide (DiSC_3_(5), Sigma-Aldrich). The bacteria were diluted to 1 × 10^7^ CFU/mL in HEPES buffer. The outer membrane of gram-negative bacteria was permeabilized by adding 0.2 mM EDTA (Sigma-Aldrich). After washing with HEPES buffer, the cells were resuspended in the same buffer containing 0.4 μM DiSC_3_(5) and 0.1 mM EDTA, and were transferred to a black 96-well microplate. The cells were incubated for 30 min under dark conditions at 37 °C to facilitate dye stabilization. Fluorescence was measured at baseline (excitation: 622 nm; emission: 670 nm) and again for 5 min immediately after treatment with the peptide [15]. An Infinite F200 Pro multimode microplate reader (Tecan, Männedorf, Switzerland) was used for every fluorescent measurement.

### 2.5. Nitric oxide (NO) Measurement and Cell Viability Assay

The cells were seeded in a 96-well microplate at a density of 5 × 10^5^ cells/mL. After 24 h, cells were treated with various concentrations of the synthesized peptide (10, 50, 100, 200, and 500 nM) and incubated overnight. The supernatants were subjected to NO measurement using Greiss reagent (Invitrogen, Carlsbad, CA, USA), and the remaining cells were used to test cell viability by employing a Quanti-Max WST-8 Cell Viability assay kit (Biomax, Seoul, Korea). The relative absorbances for the NO and cell viability assays were measured at 490 nm and 450 nm, respectively, using a microplate reader (Molecular Devices, Sunnyvale, CA, USA).

### 2.6. Reverse Transcription Quantitative Polymerase Chain Reaction (RT-qPCR)

RAW 264.7 cells were treated with the peptide with or without lipopolysaccharide (LPS, Sigma-Aldrich) for 24 h. Total RNA was isolated and 2000 ng of RNA was reverse transcribed into cDNA by M-MLV Reverse transcriptase (ELPISBIO, Daejeon, Korea). RT-qPCR was performed by employing the synthesized cDNA as well as SYBR Green PCR Master Mix (KAPA Biosystems, Wilmington, MA, USA), followed by analysis using CFX Connect™ Real-Time PCR Detection System (Bio-Rad, Hercules, CA, USA). 

### 2.7. Western Blot Analysis

The total protein content was extracted from RAW 264.7 cells using RIPA buffer (Biosolution, Seoul, Korea) with protease inhibitor cocktail and phosphatase inhibitor cocktail 2 and 3 (Sigma-Aldrich). The collected protein samples were quantified using a Pierce™ BCA Protein Assay Kit (Thermo Fisher Scientific, Waltham, MA, USA). Equivalent amounts of proteins (20 μg) were separated via 10% sodium dodecyl sulfate-polyacrylamide gel electrophoresis and transferred onto a polyvinylidene difluoride membrane. The membrane was incubated with primary antibodies after blocking with 5% skim milk (Difco Laboratories) for 1 h; subsequent washing and incubation with horseradish peroxidase-conjugated secondary antibodies followed. The separated proteins were detected using ECL Plus Western blotting detection reagents (Amersham Bioscience, Buckinghamshire, UK) using ChemiDoc™ Imaging Systems (Bio-Rad). The acquired images were analyzed and quantified using the Image Lab™ Software (Bio-Rad).

### 2.8. Statistical Analysis 

All experiments were conducted in triplicate and the results were expressed as mean ± SEM. The statistical significance of the data was evaluated by performing a one-way ANOVA test followed by Tukey’s post-test using GraphPad Prism 5.0 (GraphPad Software, La Jolla, CA, USA). *P*-values of < 0.05 were considered statistically significant. 

## 3. Results

### 3.1. Identification of Toxin Peptide from the Transcriptome of the Venom Gland of P. astrigera

**Figure 1:**
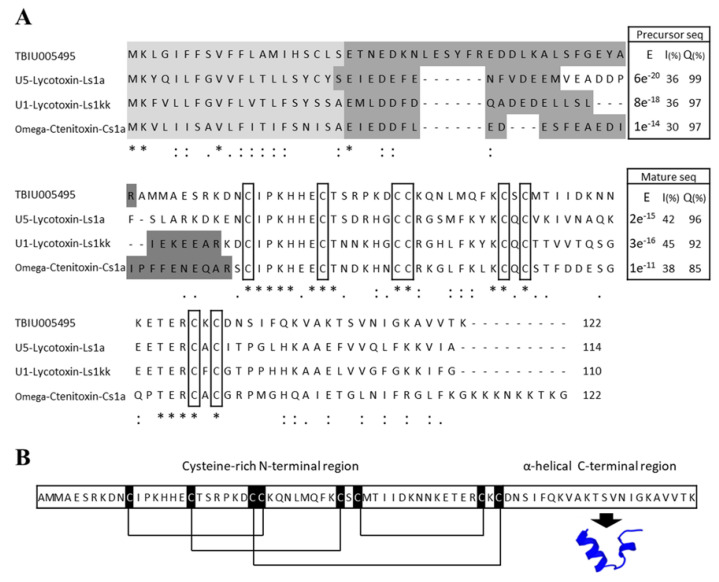
Multiple sequence alignment and structural representation of TBIU005495. (**A**) Multiple sequence alignment with other spider toxins showing significant sequence similarities with the signal peptide region (light gray), propeptide region (dark grey), and mature peptide. Conserved cysteine residues are enclosed in boxes. The symbols “*”, “:”, and “.” indicate perfect alignment, strong similarity, and weak similarity, respectively. Also, the blastP results using each precursor and mature sequence of TBIU005495 are presented with E-value (E), identity (I), and query coverage (Q). (**B**) An in silico analysis predicted that a mature sequence of TBIU005495 may have an N-terminal with disulfide bonds and a C-terminal helical domain. Disulfide bonds which were predicted by DISULFIND are known to form an ICK motif with a conserved cysteine pattern. C-terminal helix modeling was performed using the SWISS-MODEL, while purotoxin-2 was automatically selected as a template.

The transcriptome was investigated and RNA sequencing using the NGS technique was performed in search of finding a functional peptide from the venom gland of the *P. astrigera* spider. A total of 92,083,914 reads were sequenced from the sample, and the subsequent de novo assembly resulted in 149,710 transcripts. GC content was 33.95% and N50 length was 500 bp. After the construction of the venom gland transcriptome, sequences were searched against known-peptides in NCBI and Arachnoserver databases for screening sequences with >60% identity and/or 60% query coverage. The matched sequences were compared with sequences from the UniProtKB/Swiss-Prot database using the protein Basic Local Alignment Search Tool (protein BLAST, blastP) algorithm. TBIU005495 showed significant sequence similarities with the U5-Lycotoxin-Ls1a and U5-Lycotoxin-Ls1kk toxins from *Lycosa singoriensis*, and with omega-Ctenitoxin-Cs1a from *Cupiennius salei*, which exhibits selective antibacterial activity on *E. coli* mutant (Figure 1A) [16]. The SignalP and SpiderP programs were used to determine the signal and propeptide regions; toxin peptides usually contain such regions in order for them to be secreted and functionally mature. The precursor sequence of TBIU005495 consisted of a lengthy 75-mer mature peptide with a 20-mer signal sequence and a 27-mer propeptide region. Both precursor and mature sequences of TBIU005495 showed significant alignment with other peptides derived from the spider with high query coverage and low E-values. Hence, it was suggested that TBIU005495 was a spider toxin-like peptide, and a structural investigation thereof was performed.

### 3.2. Structural Characterization of the Peptide Lycotoxin-Pa4a via in Silico Analysis

Toxin peptides often have a distinguishable amino acid arrangement which forms specific motifs and has varied functions. Because TBIU005495 is a cysteine-rich peptide with eight cysteine residues, the formation of disulfide bonds was analyzed using the DISULFIND program. The result showed a distinct disulfide bond, sharing homology with other toxin peptides in multiple sequence alignments. The conserved pattern is known to form the inhibitor cysteine knot (ICK) motif, which is found in various toxin peptides that can interact with numerous biomolecules. Additionally, a number of antimicrobial peptides (AMPs) have been reported to be amphipathic and form an α-helical structure (Figure 1B) [17,18,19]. The molecular weight and net charge were predicted to be 8541 Da and +6.7, respectively. The overall results suggested the formation of an ICK motif in the N-terminal and an α-helix in the C-terminal of the TBIU005495 peptide. TBIU005495 was identified as a novel toxin-like peptide with antibacterial potential, and was named Lycotoxin-Pa4a according to the logical nomenclature of toxins [20].

### 3.3. Lycotoxin-Pa4a Shows Antibacterial Activity against Both Gram-Negative and Gram-Positive Bacteria

**Figure 2:**
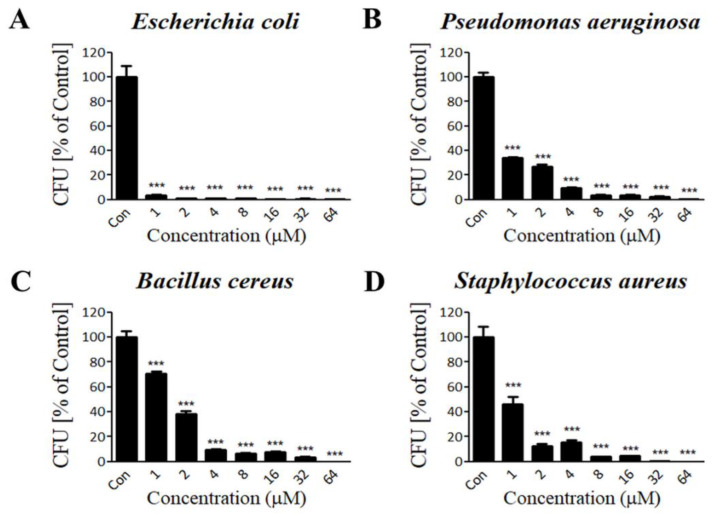
Antibacterial activity of Lycotoxin-Pa4a on (**A**) *E. coli*, (**B**) *P. aeruginosa*, (**C**) *B. cereus*, and (**D**) *S. aureus*. CFU assay was used to determine the antibacterial effect of the peptide. Results of triplicate experiments are represented as mean ± SEM. *** *p* < 0.001 indicated a significant difference compared with the control.

Lycotoxin-Pa4a was predicted to have a high positive net charge with a helical structure, which is a well-established property of AMPs. Thus, we explored the antibacterial functionality of the peptide by performing a CFU assay. Common types of pathogenic bacteria that cause infectious diseases were selected in this study. Peptide concentrations of 1, 2, 4, 8, 16, 32, and 64 μM were tested against bacterial strains. In the case of gram-negative strains, treatment with Lycotoxin-Pa4a resulted in almost complete inhibition of both *E. coli* and *P. aeruginosa* growth in a dose-dependent manner (Figure 2A,B). Similarly, gram-positive strains were also susceptible to peptide treatment. Colony formation of *B. cereus* and *S. aureus* was significantly suppressed with an increased concentration of AMP, resulting in complete inhibition at 64 μM (Figure 2C,D). The results demonstrated a strong inhibitory effect of Lycotoxin-Pa4a on both gram-negative and gram-positive bacteria, and the mechanism of action was investigated accordingly. 

### 3.4. Bacteria Membrane Permeabilization by Lycotoxin-Pa4a

The interaction of cationic AMPs with components of the cellular membrane is known to be one of the mechanisms responsible for bacterial cell death. When sufficient amounts of AMPs accumulate on bacteria, it causes pore formation and membrane disruption, causing the rapid killing of cells. A concentration of 4 μM, which induced 90% cell death on all of the strains in the CFU assay, was selected for the subsequent assays. A peptide with high antibacterial activity from the honey bee venom, melittin, was used as the positive control [21]. 

**Figure 3:**
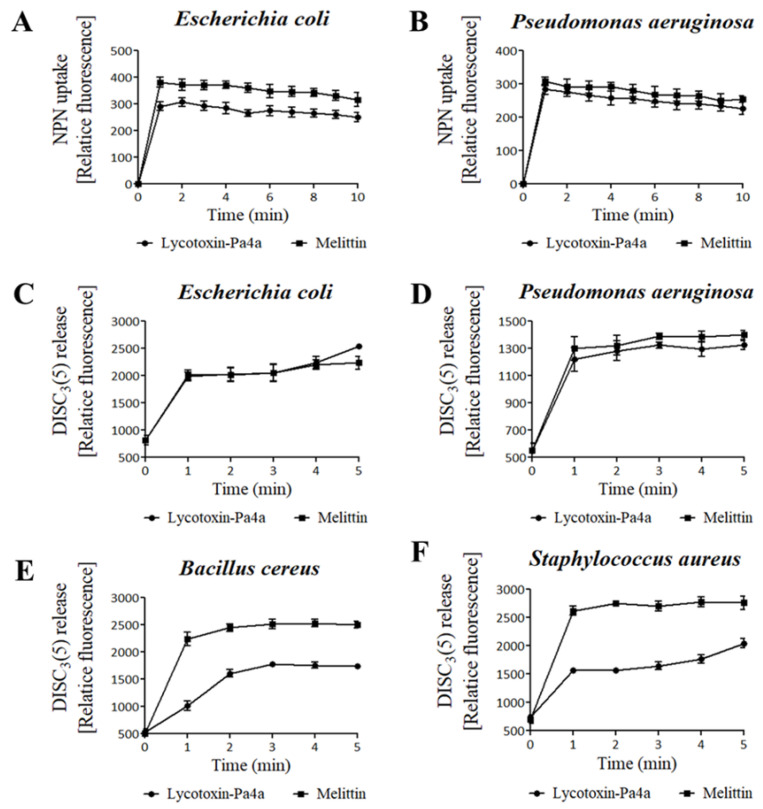
Effects of Lycotoxin-Pa4a on bacterial membrane. (**A**,**B**) NPN uptake showed outer membrane disruption by Lycotoxin-Pa4a (4 μM) in both *E. coli* and *P. aeruginosa.* The same concentration of melittin was used as control. (**C**–**F**) Depolarization of the bacterial cytoplasmic membrane was measured using DISC_3_(5) dye; 4 μM of Lycotoxin-Pa4a and melittin exposure increased fluorescence intensity for all the strains within 5 min.

First, an NPN uptake assay was performed to measure the permeabilization of the outer membrane of gram-negative strains. NPN fluoresces strong signals when it is introduced into hydrophobic conditions, and in this case, such a condition was created upon entering the inner portion of the membrane by pore formation. When fluorescence was recorded for 10 min after peptide treatment, a significant increase in the fluorescent signal was observed in both *E. coli* and *P. aeruginosa*, which was similar to or higher than the fluorescent signal generated after melittin treatment (Figure 3A,B). This demonstrated that Lycotoxin-Pa4a kills gram-negative strains by disrupting the outer membrane.

The fluorescent dye DISC_3_(5) was used to assess the destruction of the bacterial cytosolic membrane, which fluoresces when leaked out of cells by membrane depolarization. DISC_3_(5) was stabilized into the cytoplasm before peptide addition. Likewise, Lycotoxin-Pa4a treatment induced an instant spike in fluorescent signal for all the bacterial strains. Gram-negative bacteria were more vulnerable to the peptide, as the fluorescence intensity was similar to that generated after melittin treatment (Figure 3C,D). Comparably, *B. cereus* and *S. aureus* showed more rigidity towards Lycotoxin-Pa4a (Figure 3E,F). The overall results indicated that Lycotoxin-Pa4a is a toxin peptide with a strong antibacterial activity that can permeabilize both the outer membrane and the cytosolic membrane of bacteria. 

### 3.5. Lycotoxin-Pa4a Exhibits Anti-Inflammatory Effects on LPS-stimulated RAW 264.7

**Figure 4:**
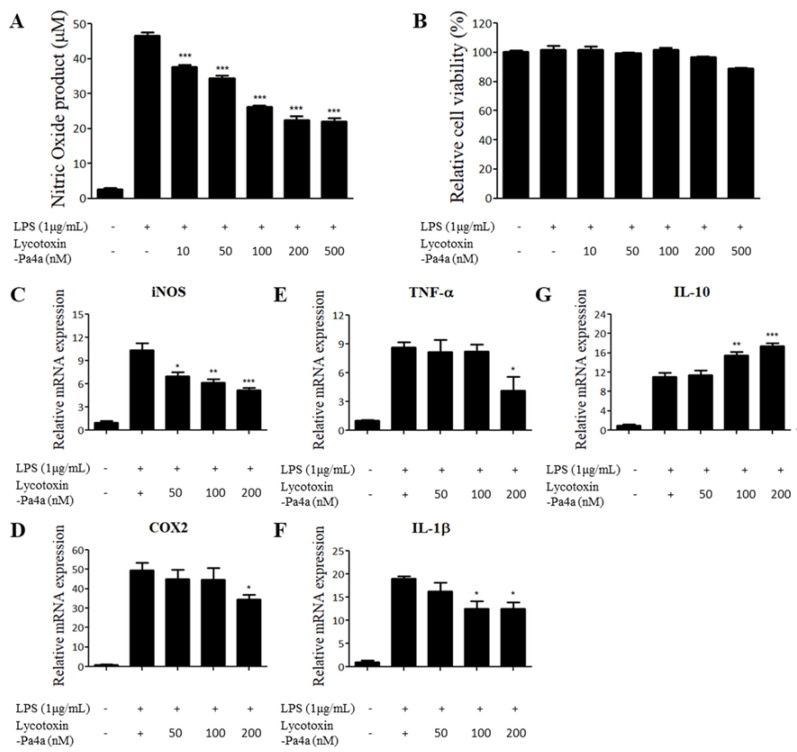
Effect of Lycotoxin-Pa4a on LPS-stimulated macrophage RAW 264.7. (**A**) Treatment of Lycotoxin-Pa4a dose-dependently decreased the production of NO up to a concentration of 500 nM. (**B**) Up to a concentration of 200 nM, cell viability remained at >90% compared with that associated with the control. (**C–F**) mRNA transcripts of the pro-inflammatory mediators, iNOS, COX2, TNF-α, and IL-1β, were suppressed by peptide treatment. (**G**) Anti-inflammatory mediator IL-10 was upregulated upon cotreatment with LPS and Lycotoxin-Pa4a.* *p* < 0.05, ** *p* < 0.01, and *** *p* < 0.001 indicated a significant difference compared with the LPS control.

AMPs are intrinsically expressed in a wide range of organisms and play a role in protecting against pathogens. Additionally, they exhibit immunomodulatory activities in innate immunity. Accordingly, we explored the anti-inflammatory effect of Lycotoxin-Pa4a on a LPS-stimulated murine macrophage model. We first examined the effect of Lycotoxin-Pa4a on cell viability and NO production on RAW 264.7 using AMP concentrations between 10 nM and 500 nM. The peptide did not show significant cell toxicity while dose-dependently decreasing the level of NO production up to a concentration of 200 nM (Figure 4A,B). Because NO plays a critical role in inflammatory responses, the changes in the mRNA expression of pro-inflammatory mediators were assessed after treatment with Lycotoxin-Pa4a via RT-qPCR. The concentrations of 50, 100, and 200 nM were selected, since high cell survival rates, i.e., over 90%, and significant inhibition of NO were shown (Figure 4A). A decrease in NO production was confirmed via downregulation of the iNOS gene (Figure 4C). The expression of COX-2, TNF-α, and IL-1β, which are major pro-inflammatory mediators, was also significantly suppressed by the peptide, with the maximum inhibition at 200 nM (Figure 4D–F). Moreover, the mRNA levels of the anti-inflammatory cytokine IL-10 were dose-dependently upregulated in the presence of the peptide (Figure 4G).

### 3.6. Inhibition of the MAPK Pathway by Lycotoxin-Pa4a

**Figure 5:**
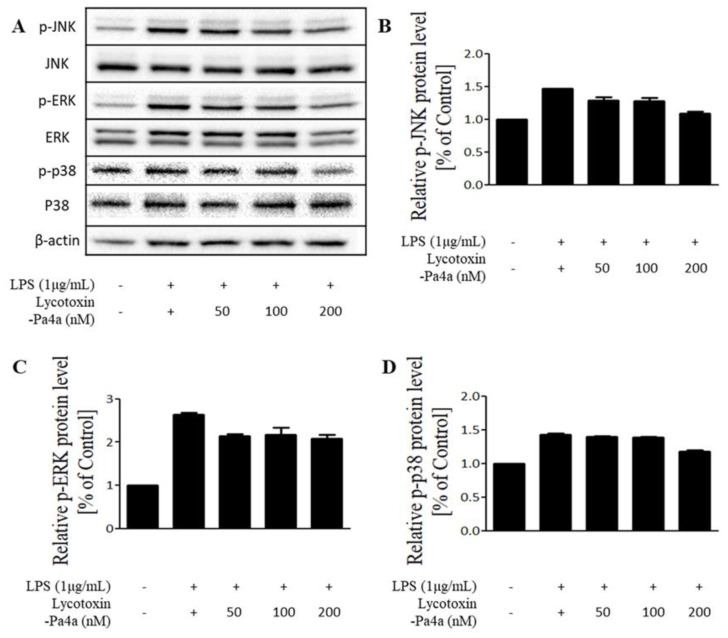
MAPK pathway was inhibited by Lycotoxin-Pa4a.Western blot showed inactivation of JNK, ERK, and p38 (**A**) and the quantified protein expression was shown (**B**–**D**).

To elucidate the mode of action for the anti-inflammatory effect of Lycotoxin-Pa4a, the molecular pathway was investigated via immunoblotting. MAPK signaling was targeted because LPS stimulation activates p38, ERK, and JNK. The phosphorylation of all proteins was inhibited by Lycotoxin-Pa4a treatment when compared with the LPS-treated sample as the positive control (Figure 5). Inactivation was observed to be dose-dependent, showing similar tendencies with the results of mRNA expression. In conclusion, Lycotoxin-Pa4a exhibited an anti-inflammatory effect along with antibacterial activity.

## 4. Discussion

The prevalence of drug-resistant microorganisms is driving the high demand for new antimicrobial reagents. AMP is known to be involved in innate immunity while being expressed across various species and having a primary antibacterial effect, along with multiple functions [22,23]. Research on the exploration and commercialization of AMP is being actively conducted, as it makes a good substitute for conventional antibiotics when taking into account its high safety, low side effects, and avoidance of drug resistance. Moreover, improvements in transcriptome and in silico analyses have accelerated the discovery of AMPs and expanded the field of exploration [24]. 

A previous study on *P. astrigera* showed the usefulness of this organism as a bio-indicator for heavy metal accumulation [25], but the identification of bioactive substances from this spider has not yet been reported. Because spider venom is a rich source of bioactive compounds, the transcriptome of the *P. astrigera* venom gland was investigated in search of a novel functional peptide. A transcript showing homology with known-toxin peptides and structural distinctiveness was identified and termed Lycotoxin-Pa4a.

Lycotoxin-Pa4a was shown to have antibacterial activity against both gram-negative and gram-positive pathogenic strains. The results of NPN uptake and DISC_3_(5) release assays revealed that the antibacterial functionality of Lycotoxin-Pa4a was induced via cell membrane disruption. Lycotoxin-Pa4a is a cationic helical peptide with a net charge of +6.7, which can interact with negatively charged LPS and peptidoglycan. The main components of the outer and cytoplasmic membranes are subjected to electrostatic force, leading to nonselective interaction between AMP and bacteria. There are well-explained models in which AMPs permeabilize bacteria, namely the barrel-stave, toroidal, carpet, and detergent models [26,27]. Disruption occurs when sufficient amounts of AMPs accumulate onto the cell membrane. AMPS can inhibit cell wall synthesis or intracellular activation by binding to molecules such as DNA, RNA, or enzymes. Additionally, AMPs are able to selectively interact with specific receptors or cellular protein components. Variance in amino acid sequences or motifs among peptides can give rise to preferential interactions with distinct microbes, leading to specific antimicrobial activity on bacteria, fungi, or viruses [28,29,30]. For instance, BilCK, ICK-containing a peptide from bumblebee, exhibits only anti-fungal activity via direct binding [31].

AMPs reportedly possess immunomodulatory activity along with the primary antimicrobial property. AMPs may not only inhibit pathogenic outgrowth, but also neutralize toxins and inhibit inflammatory responses. We tested the immunomodulatory effects of Lycotoxin-Pa4a on the LPS-stimulated RAW 264.7 model by first screening the NO production. The production of inflammatory regulator NO decreased upon costimulation by LPS and the peptide. The expression of pro-inflammatory mediators, including iNOS, COX2, IL-1β, and TNF-α, was suppressed, while anti-inflammatory cytokine IL-10 increased. As the main roles of the anti-inflammatory cytokine IL-10 are to inhibit pro-inflammatory cytokine production and recruit and activate immune cells, the peptide possesses the potential to crosslink innate and adaptive immune response. LPS stimulation leads to cytokine production and releases in macrophages via Toll-like receptor 4 (TLR-4) activation [32,33,34]. Although the mechanism of action of Lycotoxin-Pa4a remains to be determined, the downregulation of molecules involved in both acute and latent inflammation responses suggest that Lycotoxin-Pa4a can modulate the activation of TLR-4 signaling. The peptide may be involved in LPS/TLR4 interactions, since the recognition of LPS as a ligand requires multiple cell surface proteins from the macrophage. It may be considered that early activation via the MyD88 pathway, including MAPK signaling, is inhibited, as is the latent response involving the TRIF-dependent pathway [35,36]. The results suggest that Lycotoxin-Pa4a is a potent anti-inflammatory peptide that can modulate several pathways. 

Several lycotoxins are known to exhibit antimicrobial activities, including M-lycotoxin-Hc1a from the spider *Hogna carolinensis* and M-lycotoxin-Ls3a from the spider Lycosa singoriensis. These peptides share characteristics such as their amphiphilic, α-helical structure, and show antimicrobial activities via interactions with the bacterial membrane [37,38,39]. Lycotoxin-Pa4a can be distinguished from other lycotoxins by its anti-inflammatory properties. From other animal venoms, some AMPs with anti-inflammatory property have been isolated including cathelicidin-BF from the snake *Bungarus fasciatus* and melectin from the bee *Melecta albifrons* [37,38]. Further studies on the optimization and enhancement of selectivity of certain strains may produce a wider range of options for the application of peptide therapeutics [39]. Truncation or residue substitution has shown improved antimicrobial activity with lower toxicity of toxin peptides from spiders and scorpions [40,41,42]. AMPs are an ideal candidate for antibiotic development because of their ability to rapidly kill bacteria, thereby preventing the development of drug-resistant strains [43]. As the number of clinical trials and the market for peptide antibacterial agents are increasing, more focus can be directed toward peptides from biological derivatives. In conclusion, the utilization of the transcripts from a spider indigenous to Korea has led to the discovery of a novel toxin peptide with both antibacterial and anti-inflammatory activities. The results of this study suggest the potential of the toxin peptide Lycotoxin-Pa4a for use in the field of drug development, and offer insights into the functional discovery of venomous, animal-derived bioactive compounds, along with their molecular modes of action. 

## 5. Conclusions

A novel peptide Lycotoxin-Pa4a was identified from the transcriptome of the venom gland of the spider *Pardosa astrigera.* Lycotoxin-Pa4a inhibited the growth of gram-negative and gram-positive bacteria through the permeabilization of bacterial membranes. Also, the peptide modulated the expression of inflammatory mediators by inactivating the MAPK pathway. Our study demonstrated the identification of Lycotoxin-Pa4a, an antimicrobial peptide with anti-inflammatory activity, and its potential as a new source for antibiotics. 

## Supplementary Materials

The following are available online at https://www.mdpi.com/2079-6382/9/7/422/s1, Figure S1: Quality control of synthesized TBIU005495 was performed via HPLC and MS. HPLC profile (A) and MS chromatogram (B) of TBIU005495 was shown. The peptide was collected with purity >97%.
