# Supplementary material for: Antibacterial and Anti-Inflammatory Effects of Novel Peptide Toxin from the Spider Pardosa astrigera
Source: Antibiotics (Basel). 2020 Jul 19;9(7):422. doi: 10.3390/antibiotics9070422 (PMC7400607; doi:10.3390/antibiotics9070422)
Supplement: Supplementary file 1 [file antibiotics-09-00422-s001.pdf]

antibiotics

MDPI

Supplementary Materials

Antibacterial and anti-inflammatory effects of the novel peptide toxin from the spider *Pardosa astrigera*

Min Kyoung Shin [1], In-Wook Hwang [1], Yunkyung Kim [1], Seung Tae Kim [2], Wonhee Jang [1], Seungki Lee [3], Woo Young Bang [3], Chang-Hwan Bae [3], and Jung-Suk Sung [1,*]

[1] Department of Life Science, Donnguk University-Seoul, Biomedi Campus, 32, Dongguk-ro, Ilsandong-gu, Goyang-si 10326, Gyeonggi-do, Republic of Korea
[2] Life and Environment Research Institute, Konkuk University, 120, Neungdong-ro, Gwangjin-gu, Seoul, Republic of Korea
[3] Biological and Genetic Resources Assessment Division, National Institute of Biological Resources, 42, Hwangyeong-ro, Seo-gu, Incheon, Republic of Korea

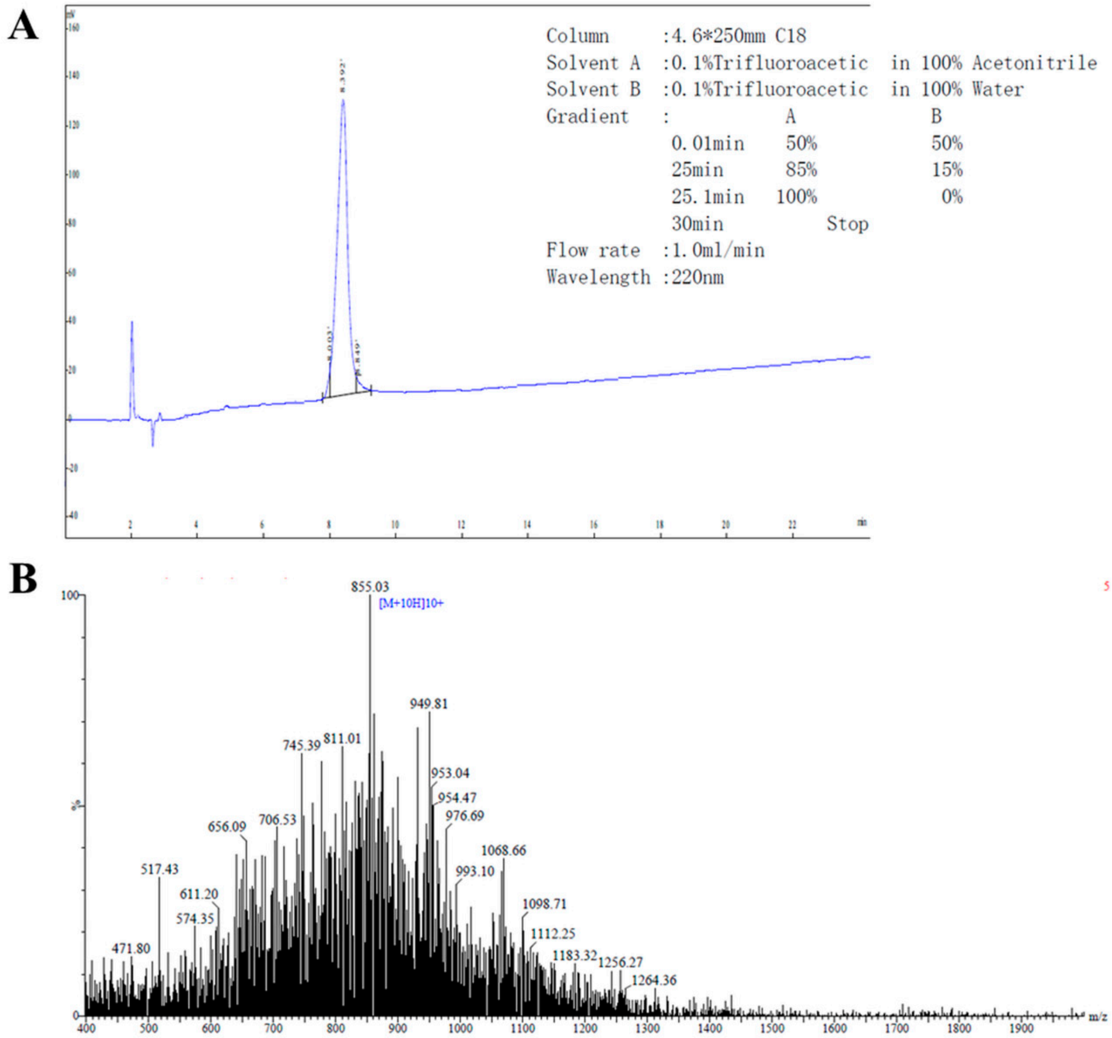

Figure S1. Quality control of synthesized TBIU005495 was performed via HPLC and MS. HPLC profile (A) and MS chromatogram (B) of TBIU005495 was shown. The peptide was collected with purity >97%.
